# 初始易瑞沙治疗有效的晚期肺腺癌二次使用易瑞沙的疗效分析

**DOI:** 10.3779/j.issn.1009-3419.2012.01.09

**Published:** 2012-01-20

**Authors:** 峻岭 李, 学志 郝, 燕 王, 湘茹 张, 远凯 石

**Affiliations:** 100021 北京，中国医学科学院，中国协和医科大学肿瘤医院内科 Cancer Institute and Hospital, Chinese Academy of Medical Sciences and Peking Union Medical College, Beijing 100021, China

**Keywords:** 肺肿瘤, 易瑞沙, 再次使用易瑞沙, Lung neoplasms, Iressa, Readministration of iressa

## Abstract

**背景与目的:**

吉非替尼（商品名易瑞沙）是表皮生长因子受体酪氨酸激酶抑制剂，现广泛应用于晚期非小细胞肺癌。易瑞沙对于女性、腺癌、非吸烟者、亚洲人的疗效较好。已有一些临床研究发现使用易瑞沙临床获益的患者疾病进展后，再次使用易瑞沙可能会获益。本研究旨在评估二次使用易瑞沙的疗效及安全性。

**方法:**

回顾性分析我院18例初始使用易瑞沙临床获益的晚期肺腺癌患者治疗失败后二次使用易瑞沙治疗的疗效。

**结果:**

二次易瑞沙治疗后，1例患者获得部分缓解，11例患者获得疾病稳定，6例患者疾病进展。疾病控制率为67%，中位无进展生存为5.16个月。初次使用易瑞沙的中位生存时间为39.4个月，二次使用易瑞沙的中位生存时间为12.41个月。不良反应可以耐受。

**结论:**

初次使用易瑞沙有效的晚期肺腺癌患者，二次使用易瑞沙仍有可能延长患者的生存时间。

## Abstract (en)

**Background and objective:**

Gefitinib is an epidermal growth factor receptor-tyrosine kinase inhibitor (EGFR-TKI) that has been widely used for the treatment of non-small cell lung cancer (NSCLC). It is most effective in women, as well as in patients who have never smoked, have pulmonary adenocarcinomas, or are of Asian origin. Several treatment options are available for NSCLC patients who responded to initial gefitinib therapy but demonstrated tumor progression, of which gefitinib readministration is the chosen therapeutic option. The present study aims to evaluate the efficacy and toxicity of gefitinib readministration.

**Methods:**

The clinical data of 18 patients with NSCLC who had shown partial response (PR) or achieved a stable disease (SD) status after gefitinib administration and were retreated with gefitinib due to failure of the initial therapy were reviewed and retrospectively analyzed.

**Results:**

Of the 18 patients studied, 1 (6%) showed partial remission (PR), 11 (61%) achieved SD, and 6 (33%) experienced disease progression. The disease control rate was 67%, and the median progression-free survival was 5.16 months (range, 1 to 24.8 months). The median overall survival from the start of the gefitinib therapy was 39.4 months (range, 15.38 to 52.44 months). Moreover, the median overall survival from the beginning of the 2nd therapy was 12.41 months (range, 3.98 to 38.24 months). Mild toxicity was observed with the 2^nd^ gefitinib therapy.

**Conclusion:**

The results of the present study indicate that patients with NSCLC may still be expected to achieve prolonged survival through gefitinib readministration if they initially responded to gefitinib and underwent various subsequent treatments.

晚期非小细胞肺癌含铂方案化疗可延长生存期，改善症状，然而目前化疗疗效已经达到一个平台，新药联合铂类的总体有效率25%-35%，中位生存期仅8个月-10个月，且仅适合于PS评分较好的患者，对于PS评分较差（3分-4分）者，化疗不能使患者获益，且毒副反应明显。表皮生长因子酪氨酸激酶抑制剂（epidermal growth factor receptor-tysine kinase inhibitors, EGFR-TKIs）吉非替尼和厄洛替尼是作用于表皮生长因子受体酪氨酸激酶，阻断信号传导，从而抑制肿瘤生长的小分子靶向药，口服用药，使用方便且毒副反应轻微。EGFR-TKIs对于部分选择患者疗效明显^[1-3]^，选择性因素包括亚裔、女性、非吸烟、腺癌。IPASS^[4]^研究中吉非替尼一线用于亚裔非吸烟腺癌患者优于紫杉醇联合卡铂方案化疗。NCCN指南中明确指出对于PS评分3分-4分，*EGFR*突变的患者可使用厄洛替尼或吉非替尼（1类共识）。

然而，尽管许多患者初始EGFR-TKIs治疗疗效显著，绝大多数最终会复发。并且在中国，多数患者在二、三线治疗时才开始使用EGFR-TKIs治疗，进展后可选择的治疗有限，且疗效欠佳，有报道^[5]^二、三线化疗的有效率仅6.8%，由于已行多程治疗，患者对化疗的耐受性也显著下降。关于二次使用EGFR-TKIs的几项研究^[6-8]^的结果提示初始治疗有效的患者二次使用EGFR-TKIs可能有效。

本研究旨在通过回顾性分析我院接受二次易瑞沙治疗的患者的资料，了解二次TKIs的疗效，并进一步探讨可能的机制。

## 1 材料与方法

回顾性分析中国医学科学院肿瘤医院自2001年1月-2011年7月就诊的晚期非小细胞肺癌患者资料，有18例初始易瑞沙治疗临床受益[部分缓解（partial response, PR）或疾病稳定（stable disease, SD）]的患者接受了2次易瑞沙治疗。治疗持续至肿瘤进展或不能耐受的毒副反应。疗效评价采用RECIST实体肿瘤评价标准。无进展生存（progression free survival, PFS）定义为治疗开始至肿瘤进展的时间，总生存期（overall survival, OS）为治疗开始至患者死亡时间。使用SPSS软件进行数据分析。

## 2 结果

### 2.1 患者特征

共18例患者，初始易瑞沙有效，二次使用易瑞沙，男性7例，女性11例，年龄37岁-74岁，中位年龄50.5岁。病理类型18例均为腺癌。2例患者易瑞沙为初始治疗，其余患者均在易瑞沙治疗前接受过含铂方案化疗。患者基线特征见表 1，患者的治疗总结见表 2。

**1:** 患者基线特征 Basic characteristics of all patients

	*n*	%
Total	18	
Gender		
Male	7	38.9
Female	11	61.1
Age (yr)		
Median	50.5	
Range	37-64	
Smoking history		
Never	14	77.8
Ex-smoker	1	5.6
Current	2	11.2
Unknown	1	5.6
Histological type		
Adenocarcinoma	18	100
*EGFR* mutation		
Exon-19	1	5.6
L858R	0	0
Unknown	17	94.4
EGFR: epidermal growth factor receptor.		

**2:** 所有患者的治疗情况 The clinical course of all the patients

	Age (yr)	Gender	Smoking history	Histological type	*EGFR* mutation	PFS-1^st^TKIs (month)	Interval of TKIs	The chemotherapy of the interval	KPS	Response	PFS-2^nd^TKIs (month)	OS-2^nd^TKIs (month)
1	68	Male	Never	Adeno	-	10.00	10.41	GEM+CBP	-	PR	1.61	23.03
2	55	Male	Never	Adeno	-	21.26	7.82	Pem	-	SD	3.25	22.00
3	49	Male	Current	Adeno	-	22.21	3.00	Pem+DDP	90	SD	8.00	26.71
4	43	Female	Never	Adeno	-	8.77	5.00	TXT+endo	70	/	1.00	6.00
5	37	Female	Never	Adeno	-	14.09	5.42	Pem+DDP	90	PD	8.02	12.48
6	49	Female	Current	Adeno	-	6.00	4.00	PTX+IFO	80	SD	1.00	10.00
7	63	Female	Never	Adeno	-	30.0	4.00	Pem+CBP,	-	SD	Till now	15.70
8	47	Male	Ex-smoker	Adeno	-	18.37	6.31	PTX+NDP	-	PR	5.32	12.35
9	70	Female	Never	Adeno	-	22.00	3.00	PTX+DDP	70	PD	19.00	21.00
10	46	Male	Never	Adeno	-	Cease for rash	9.00	No treatment	-	/	14.00	17.00
11	41	Female	Never	Adeno	-	10.28	4, .60	Pem+DDP	-	SD	3.00	6.37
12	71	Male	Never	Adeno	-	10.35	13.00	Pem+DDP	90	SD	1.00	9.00
13	52	Female	Never	Adeno	-	16.00	10.00	BIBW2992	-	/	5.00	5.00
14	56	Female	Never	Adeno	-	7.59	2.14	PTX+IFO	90	SD	24.80	38.24
15	38	Female	Never	Adeno	-	9.00	23.00	PTX+CBP; TXT	-	PR SD	9.00	9.00
16	74	Male	Never	Adeno	-	Cease for rash	22.00	Chinese medicine		/	13.00	17.00
17	48	Female	Never	Adeno		24.97	23.00	AZD2171		/	Till now	4.47
18	72	Female	Never	Adeno	Exon-19	7.03	4.37	Pem	80	SD	4.30	3.98
Adeno: adenocarcinoma; PR: partial response; SD: stable disease; PD: progressive disease.												

### 2.2 初始易瑞沙治疗的疗效

初次行EGFR-TKIs治疗时，有12例患者获得了部分缓解（70%），6例患者疾病稳定（30%）。中位无进展生存为12.22个月（范围：4个月-13个月）。其中2例患者分别在服用易瑞沙1个月及4个月时因皮疹自行停药，前者疗效SD，后者最佳疗效PR。

### 2.3 二次易瑞沙治疗的疗效

14例患者初始易瑞沙失败后接受了细胞毒药物化疗，1例患者未治疗，1例患者中药治疗，2例患者分别接受BIBW2992及AZD2171临床试验药物治疗。两次TKIs治疗的时间间隔在2个月-13个月，中位时间5.21个月（表 3）。1例（6%）患者PR，11例（61%）患者维持稳定，6例（33%）患者PD。疾病控制率（disease control rate, DCR）达67%。中位PFS为5.16个月。第一次TKIs治疗的中位OS为39.4个月（范围：15.38个月-52.44个月），第二次TKIs的中位OS为12.41个月（范围：3.98个月-38.24个月）。

**3:** 易瑞沙治疗前的治疗情况 Summary of prior therapy

	*n*	%
No. of chemotherapy regimens before gefitinib		
0	2	11.1%
1	11	61.1%
2	5	27.8%
Best response to gefitinib		
PR	12	66.7%
SD	6	33.3%
Interval from discontinuation of gefitinib to 2^nd^ EGFR-TKIs		
Median (95%CI) (month)	5.86 (5.42-12.36)	
PFS-1^st^TKI		
Median (95%CI) (month)	12.22 (10.93-18.81)	
PFS-2^nd^TKI		
Median (95%CI) (month)	5.16 (4.44-11.27)	

### 2.4 两次易瑞沙之间的治疗情况

两次TKIs治疗期间，1例患者未治疗，1例患者中药治疗，2例患者入临床试验组，分别接受AZD2171及BIBW2992的治疗，余14例患者接受化疗，中位化疗周期数为4周期（2个-16个周期），13例可评价患者中，有效率（PR）达23.1%，疾病控制率（PR+SD）为84.6%。

### 2.5 治疗后疾病进展的部位的比较

初次EGFR-TKIs治疗后2例因不良反应（皮疹）终止治疗，其余16例患者均出现进展，其中肺原发灶进展者6例（33%），肺内转移进展者10例（56%），胸膜病变进展者4例（22%），位于骨、肝、脑及肾上腺者分别为2例、1例、1例、1例。二次治疗后2例患者至随访终止时病情仍稳定，3例患者病历资料欠缺，其余15例患者中3例（20%）出现原发灶进展，9例（60%）位于肺内转移，2例胸膜病变（13%），3例脑转移（20%）。

### 2.6 安全性

易瑞沙的耐受性较好。初始易瑞沙治疗与再次易瑞沙治疗的不良反应均较轻微，表现为1度-2度的皮疹，轻度的腹泻。有2例患者在初始治疗中因皮疹停药。

## 3 讨论

我们的研究中初次易瑞沙治疗临床获益的患者，经过一段时间二次接受易瑞沙治疗仍有67%的患者可获益，再次验证了既往的研究结果^[6, 8-10]^。关于易瑞沙治疗后二次获益的具体机制还不清楚。不同的研究提出了多个解释。有报道^[11, 12]^体外实验证明某些细胞毒药物可以通过增加EGFR磷酸化水平使非小细胞肺癌肿瘤细胞重新获得对易瑞沙的敏感性，两次易瑞沙治疗间的化疗有可能降低易瑞沙耐药的肿瘤细胞比例。然而，发表在*Clinical Cancer Research*上的一项研究^[13]^结果显示，转移性非小细胞肺癌患者行EGFR-TKIs（6例易瑞沙、7例厄洛替尼治疗）治疗后进展，停用药物后3周（期间不进行其它抗肿瘤治疗）再次使用TKIs，10例患者中7例肿瘤的直径保持稳定，8例患者肿瘤体积缩小。因此，上述理论似乎不能完全解释此现象。

*EGFR*二次突变被认为与TKIs耐药相关。获得性耐药的患者中50%存在T790M突变^[14-16]^，20%存在*MET*基因扩增^[17]^。T790M突变使患者对易瑞沙或厄洛替尼治疗抗拒，然而有研究认为获得性耐药的肿瘤组织中仅一小部分存在T790M突变，部分肿瘤对TKIs治疗仍有效^[18, 19]^。因此，获得性耐药的患者继续使用TKIs类药物仍有可能获益。我们的研究发现初次病变进展的部位与二次TKIs治疗进展的部位往往相同，或许可以用该理论予以解释。此外，二次突变中亦可能产生药物敏感性突变，如，L858R或外显子19缺失突变等，也可能是二次TKIs治疗有效的原因。临床研究^[20]^表明厄洛替尼对于吉非替尼非优势人群亦有效。厄洛替尼的血药浓度明显高于吉非替尼^[21, 22]^，首次使用易瑞沙进展后，使用厄洛替尼可能会使疗效增加。此外，体外研究^[8]^显示非T790M突变对EGFR-TKIs部分抗拒，明显低于T790M突变的耐药性。增加血药浓度亦有可能克服非T790M突变造成的耐药。

总之，EGFR介导的信号传导通路有多个，下游信号分子亦很多，通路中的任何一个部位的改变都有可能造成药物敏感性的改变，关于TKIs二次治疗的效果及机制还需要进一步的研究来明确。

我们的研究提示初始易瑞沙治疗有效的患者，在治疗失败后，再次使用易瑞沙仍可获益。这为非小细胞肺癌患者的治疗提供了非常有价值的信息。目前已有研究均为小样本的回顾性分析，有待进一步的前瞻性研究来证实。
